# A Novel and Versatile Microfluidic Device for Cell Assays under Radio Frequency Exposure

**DOI:** 10.3390/bios13080763

**Published:** 2023-07-27

**Authors:** Mengshuang Wang, Mengni Zhu, Zhenjie Zhao, Xin Li, Jie Zhang

**Affiliations:** Shanghai Key Laboratory of Magnetic Resonance, Engineering Research Center for Nanophotonics & Advanced Instrument, Ministry of Education, School of Physics and Electronic Science, East China Normal University, Shanghai 200241, China

**Keywords:** cell mobility, radiofrequency electromagnetic field, microfluidic chip

## Abstract

Wound healing is a complex process composed of different stages, which involves extensive communication between the different cellular factors of the extracellular matrix (ECM). The radio frequency electromagnetic field (RF-EMF) has been used to accelerate the wound-healing process and it has been found to enhance cell alignment and mobility. The conventional methods for cell mobility analysis in an electromagnetic field generated by a radiation source are not advisable due to the low-precision, nonuniform distribution of the field, low efficiency of the analysis in batch and the lack of system integration for autonomous on-body operation. Here, a novel and versatile electromagnetic exposure system integrated with a microfluidic chip was fabricated to explore the EMF-induced response. A gradient electromagnetic field in a two-dimensional plane has been successfully established in the microchambers placed along the field line. In this work, by deploying our radiation experiments in vitro, we validated the on-chip monitoring of cell response to exposure. This electromagnetic field was simulated and human amniotic epithelial cells (HAECs) were cultured in different microchambers for continuous exposure to the electromagnetic field excited by a monopole RF antenna (1.8 GHz). New protrusions were generated and an obvious increase in filopodia with the increased field intensity was investigated. Meanwhile, the variation in intracellular Ca^2+^ concentration under the electromagnetic field was examined. The inhibitory effect of the Ca^2+^ circulation was further inspected to reveal the potential downstream signaling pathway in the RF-EMF-related bioassay, suggesting that cytoskeletal dynamics of cells under exposure are highly associated with the EGF receptor (EGFR)-cytoskeleton downstream signaling pathway. Finally, the field-induced cell elongation and alignment parallel to the field direction were observed. Additionally, the subsequent recovery (field withdrawal) and re-establishment (field re-exposure) were explored. These results indicated that this reliable and versatile exposure system for bioassay could achieve precise and high-throughput detection of the RF-EMF-induced cytoskeletal reorganization in vitro and evaluate the possible health risk from RF-EMF exposure.

## 1. Introduction

Wound healing caused by injuries that occur following physical, chemical, or thermal insults is a complex process, as the disruption of the skin and the subsequent breakdown of the barrier protecting the human body may induce inflammatory, proliferative and immune reactions [1]. The wound-healing process is composed of many phases, including hemostasis, inflammation response, cell mobility, new tissue formation and tissue remodeling phases [2,3,4]. Pervious work showed that a radio frequency electromagnetic field (RF-EMF) can accelerate the wound-healing process [5]. The RF-EMF is non-ionizing energy at the short-wave radiofrequency band of the electromagnetic spectrum that may be used as a non-invasive therapy delivered through a wound’s dressing using small and portable devices that can be self-applied [6]. Researchers found that it may affect nitric oxide signaling, the modulation of cytokine profiles, the expression of growth factors and the regulation of mitogen-activated protein kinase/extracellular signal-regulated kinase, resulting in the wound healing [7].

Nowadays, an interest focused on wound healing when exposed to the RF-EMF radiation has led to more and more investigation [8,9]. These studies to accelerate wound healing and tissue reorganization after an injury are crucial, and highlighted the new therapeutic perspective of using EMF radiation to promote wound healing. In fact, several assays have confirmed that the EMF facilitates wound healing by guiding cell migration [10]. Basically, among these, the in vitro scratch assay was preferential to test if there was quantifiable migration when exposed to the RF-EMF radiation [11]. However, it was not a method of choice if the availability of cells (e.g., specialized primary cells that are hard to obtain in sufficient amounts) or chemicals (e.g., expensive reagents) was limiting [12]. Meanwhile, the development of exposure systems for cell assay investigation is rapidly spreading. In recent years, various exposure systems have been designed, such as reverberation chambers [13,14], the pair of coils and the waveguide [15,16], to evaluate exposure-associated cellular response through Specific Absorption Rate (SAR). Not taking into account the relation between EMF and cell migration, SAR usually provides a widely accepted description of RF-EMF-induced responses [17]. Indeed, wound healing involves morphological responses, including the actin cytoskeleton shifting from one state to another, behaving in newly formed protrusion patterns, changes in cellular shapes and the beginning of movement such as elongation, alignment, or even migration [18], which are closely related to the uniformity and orientation of field at the given location when cells are cultured in chamber-like conventional flasks [19], culture plate wells, or Petri dishes [20,21]. These above-mentioned systems fail to achieve a precise assessment in terms of the morphological responses. Furthermore, the aforementioned exposure chambers tend to consist of bulky field generators such as multi-shaped waveguides [15], pillar antennas, or a pair of Helmholtz coils [13]. These generators are hard to operate. Therefore, a well-designed exposure system is required to generate a controllable EMF for effective and efficient exploration of the cell response to RF exposure. Microfluidics, a multifunctional and burgeoning technology, has shown compelling advantages over traditional approaches [22,23], including design-of-experiment [24], precisely controlled manner [25], high-throughput analysis and low consumption of reagent [26]. Hence, it is believed that microfluidic devices could establish a well-controlled stimulus to solve the existing aforementioned barriers and investigate the cell mobility response caused by RF-EMF.

In this work, a novel and versatile electromagnetic exposure system integrated with a microfluidic chip was designed and fabricated to explore the cell responses when exposed to 1.8 GHz RF-EMF (0.5 h) which can overcome the limitations of previously investigated exposure systems. To render operability, we integrated the cell effect detection interface into a miniaturized custom-developed radiation platform, equipped with the designed micro-chip, wireless and programmable radiation excitation and an automatic syringe pump. To validate the utility of our system for radiation applications, we utilized it to monitor the cell response to RF-EMF in vitro. The morphology of cells cultured in different chambers on the chip was investigated. It was also found that the morphology of cells cultured in different chambers on the chip and the intracellular Ca^2+^ concentration have been investigated. Additionally, two specific inhibitors, calcium channel inhibitor (Nifedipine) and tyrosine kinase inhibitor (PD), were utilized to reveal the EMF-related responses. The results confirmed that all morphological responses induced by exposure were related to the EGFR-cytoskeleton downstream signaling pathway. The morphological differences of cells under different exposure conditions were also found, including exposure, EMF withdrawal and re-exposure. Basically, whether exposed or re-exposed, with the increase in field strength, cell orientation tended to elongate and align along the field direction. Subsequently, this cell alignment trend disappeared when the EMF was withdrawn; these results suggest that the influence of the RF-EMF is not likely permanent but is, more likely, recoverable. In this work, this easy-operated exposure system has been established to achieve the precise inspection and high-throughput analysis of the variation in cell morphology induced by RF-EMF, in the hope of further revealing localized cell responses to the field exposure.

## 2. Materials and Methods

### 2.1. Fabrication of Microfluidic Chip

Device fabricated using soft lithography in this study was previously described and is shown in Figure S1 [27]. The microfluidic channel was made of Polydimethylsiloxane (PDMS, RTV-615, Momentive, Shanghai, China) using soft lithography with an SU-8 (MicroChem, Newton, MA, USA) patterned wafer. A monopole antenna copper wire (41.67 mm in height and 0.17 mm in radius) was inserted into PDMS (10:1) before curing at room temperature for 72 h for the integration of the monopole antenna on the chip, which also enabled the insulation of the antenna from the culture media. After curing, the PDMS casting (width ∼45 mm, length ∼45 mm, thickness ∼5 mm) was peeled off from the mold. After manual alignment and O_2_ plasma treatment, the PDMS casting was bounded with glass substrate with a hole in the center for cabling. Fabricated channels were dried at 70 °C for at least 1 h. The punched ground electrode was placed on bottom of the microdevice. Finally, the monopole antenna copper wire soldered with the cable on the bottom of the antenna was inserted into the central hole. Before cell seeding, the microfluidic device needed to be sterilized via UV irradiation for 30 min. Notably, the microfluidic device was placed in the incubator to maintain the cell viability. 

### 2.2. Simulation and Characterization of Radiation Field

The characteristics of the RF-EMF, including direction, intensity and uniformity, were simulated. This simulation was based on two-dimensional RF-EMF parameters around the cells cultured in micro-chambers to facilitate the observation and result analysis of cell morphology response. Meanwhile, to validate the repeatability and reliability of the exposure system designed, the EMF simulation system was implemented in different environments, including only monopole antenna, devices embedding the PDMS and culture media added into microfluidic chambers. The simulation and performance test results include the performance test, like return loss (RL), were tested using a Vector Network Analyzer (Keysight technology, Rosa, CA, USA; N5247B). In theory, according to the results of RL, the value of Voltage Standing Wave Ratio (VSWR) can be calculated based on Equation (1).

(1) $$\mathrm{RL}=20{\mathrm{Log}}_{10}(\frac{\mathrm{VSWR}+1}{\mathrm{VSWR}-1})$$

### 2.3. Cell Culture and Preparation

The HAECs were cultured in accordance with East China Normal University guidelines and maintained using a previously reported method [28]. HAECs were purchased from IMMOCELL (Xiamen, Fujian, China). Firstly, HAECs were cultured in Petri dish through the Minimum Essential Medium (MEM), which was supplemented with 10% BSA and 1% Penicillin-streptomycin. The cells were detached with 0.25% trypsin/EDTA at 80% confluence, followed by centrifugation (1000 rpm, 5 min) and resuspension. A solution of fibronectin (FN) at 100 μg/mL concentration (Sigma, Shanghai, China) in 0.1 M NaHCO_3_ (pH = 8) was used to coat the channels and the chambers of the device at 37 °C for 1 h. Hereafter, 50 μL HAEC suspension (10^6^/mL) was loaded into the microchannels by aspiration through a syringe pump (neMESYS 290N, CETONI, Korbußen, Germany) at a low flow rate. To ensure the nutrient exchange for the HAECs grown in the microfluidic device, the medium was perfused every 2 h at a rate of 1 μL/min for 15 min into the microchannels. The cells in micro-chamber were maintained in an incubator (5% CO_2_ at 37 °C) for 24 h to attain a desired cell morphology and density. All groups of cells were starved for 12 h in serum-free medium prior to experiments to synchronize the cells. Additionally, the cells were used in experiments at passage 4 or 5. All experimental operations were strictly performed under sterile conditions.

### 2.4. Immunostaining and Imaging

Cytoskeleton staining: After RF exposure, the cytoskeleton of HAECs was stained using Phalloidin-TRITC. Briefly, the cells seeded in chip were starved for 12 h in serum-free medium prior to experiment. We gently washed cells with PBS twice. The cells were fixed with 4% formaldehyde in PBS for 20 min on the ice. Gently washed cells with PBS three times again. Cells were permeabilized with 0.1% Triton X-100 in PBS for 10 min at room temperature. Gently washed cells with PBS three times again. Added 1 mL (2 mg/mL) Phalloidin-TRITC staining solution into microchannels, and afterward cells were stained for 30 min at room temperature. Gently washed cells with PBS three times. Finally, added 1 mL (100 nM) DAPI into microchannels to stain the nucleus. The fluorescence images of cytoskeleton reorganization were captured using a DMi8 Inverted Microscope (Leica, Wetzlar, Germany; X400).

Cytosolic Ca^2+^ staining: The cytosolic Ca^2+^ concentration in HAECs was analyzed using Fluo-4, AM/DMSO solution. Briefly, the HAECs were starved for 12 h in serum-free medium containing 10 % CaCl_2_ (0.2 mM) prior to experiment. We gently washed cells with PBS twice, added 1 mL (4 μM) Fluo-4, AM/DMSO staining solution supplemented with Pluronic F127 into microchannels, then incubated at 37 °C for 20 min. Added 5 mL HBSS containing 1% fetal bovine serum and continued to incubate for another 40 min. Gently washed the cells three times with HEPES buffer saline. Eventually, added 1 mL prepared DAPI into microchannels to stain the nucleus. The fluorescence images of cytosolic Ca^2+^ concentration of HAECs were detected using a Confocal Laser Microscope (Nikon-Ti E; X400).

The inhibitory effect assay: At first, the seed and culture of HAECs on the chip followed the aforementioned methods. Secondly, cells were starved in serum-free medium when they covered 85% of each chamber to synchronize the cells. 12 h later, cells were pre-treated with PD (2 h), Nifedipine (40 min), or their combination. Then, the cells were continuously exposed to RF-EMF (1 W, 1.8 GHz) for 0.5 h. After RF-EMF exposure, they were gently rinsed with PBS 3 times for 10 min and fixed immediately in 4% paraformaldehyde at room temperature (10 min). Subsequently, the cells were rinsed again with PBS and permeabilized with 0.22% Triton-100 for 10 min. Afterwards, the cells were washed again in PBS. Eventually, the effects of inhibitors were evaluated with the results of immunofluorescence staining of HAECs. A sham group was also performed as control. The cells in the microchambers were incubated for 30 min without the electromagnetic radiation. After incubation, the cells were analyzed according to the same treatment protocol as the radiation group.

## 3. Results and Discussion

### 3.1. Design and Fabrication of Exposure Microsystem and Operational Principle

**Figure 1:**
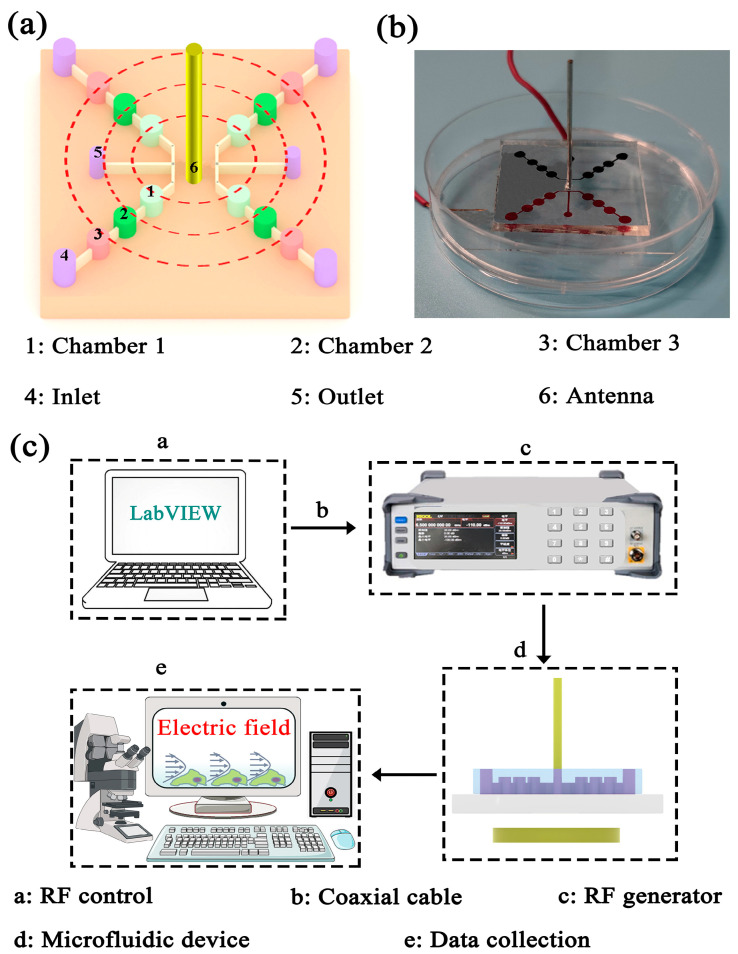
(**a**) The schematic of the micro-chambers. (**b**) The insert shows a photograph of the micro-chambers. (**c**) Schematic illustration of electromagnetic exposure system.

The fabrication process for the microfluidic channel is presented in Figure S1 (Supporting Information). Figure 1a shows the schematic of the micro-chambers. In brief, the microfluidic device was composed of four outlets, two inlets and four branch channel units, in which the two branch channels on one side were connected and shared one inlet (Figure 1a). Each channel contained three chambers (50 µm in height and 1400 µm in radius) and the chambers were interconnected by the guided channel (width ∼400 μm, height ∼50 μm), which was pre-designed on the micro-chip to identify the EMF direction. A photograph of the microfluidic device is shown in Figure 1b, which indicates the possibility of the precise and high-throughput detection of the RF-EMF-induced cytoskeletal responses. 

Figure 1c shows the overview of the exposure system and the operational principle. This microfluidic exposure system was composed of three main parts: The RF generator (1.8 GHz), the power amplifier and the microfluidic device. Each part was connected with lossless coaxial cable. Firstly, a computer was programmed to control the parameters of a radiation field, like frequency and waveform, in real time through LabVIEW. The generated RF signal was amplified, converted and transmitted to the monopole antenna on the chip. A uniform RF-EMF could be generated and continuously stimulate the HAECs in the chamber. Eventually, all results were collected by a user interface.

According to the schematic illustration of the culture system (Figure S2, Supporting Information), two side channels were connected to the perfusion system. The injection pump filled the micro-chambers with cells by means of negative pressure injection. The culture system can achieve high throughput and automation.

### 3.2. Numerical Simulation

**Figure 2:**
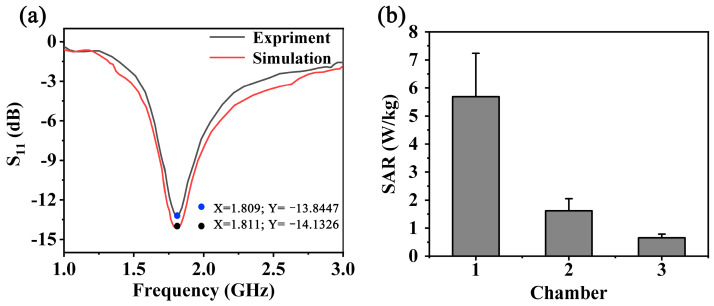
The simulation results of the monopole antenna. (**a**) The return loss results of the simulation and experiment. (**b**) The average SAR value of the three chambers exposed to RF-EMF.

In this work, the two-dimensional distribution of the EMF excited by the monopole antenna (λ/4) was simulated with high-order and accurate analysis. The relevant results of the simulation model are presented in Figure 2. As shown in Figure 2a, the VSWR values of the numerical simulation and experiment can reach 1.49 and 1.56, respectively. The direction of the two-dimensional RF-EMF was aligned along the guided channel rather than in a random direction. In addition, the assessment of the SAR was unignorable when exposed to RF exposure, which means the energy was absorbed [29]. The corresponding results are presented in Figure 2b, in which the average two-dimensional SAR values of three chambers are located at 5.69 W/Kg, 1.61 W/Kg and 0.66 W/Kg, respectively. During the simulation of the field, we selected different positions of the chamber, in which position was uniformly selected along the guided channel (Figure S3, Supporting Information). According to the simulated EMF intensity results, the average field values of the three chambers in the *xy* plane can reach 43.2 V/m, 27.3 V/m and 17.8 V/m, respectively. 

### 3.3. The Cytoskeleton Reorganization of HAECs under RF-EMF

**Figure 3:**
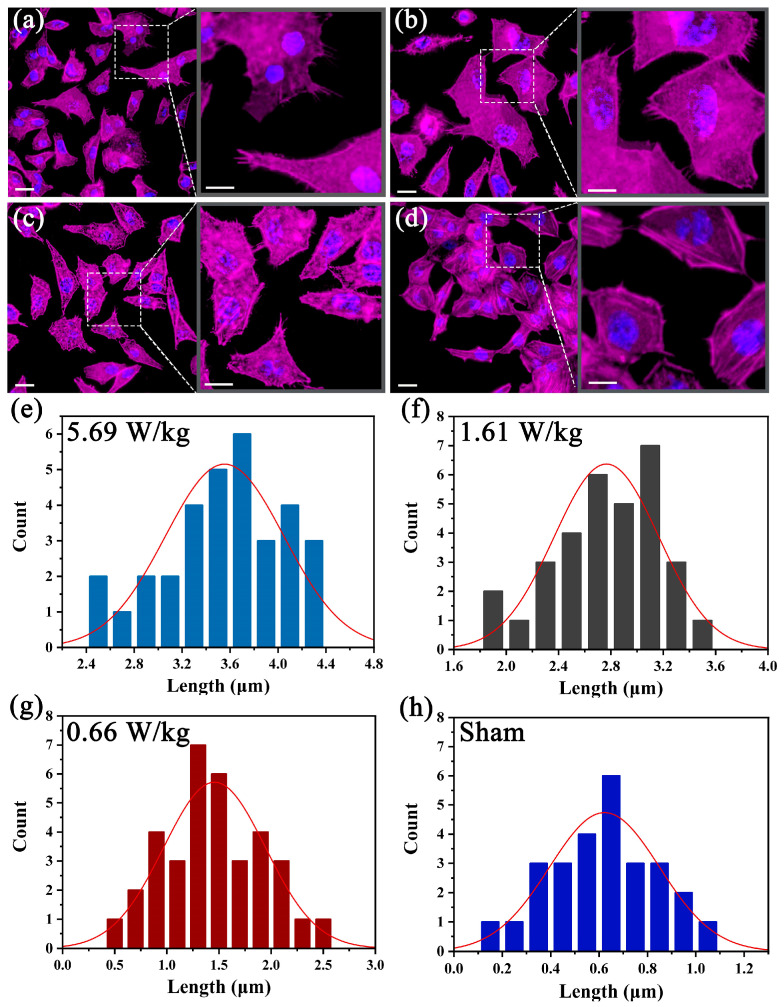
RF-EMF exposure induces filopodia-like protrusions in HAECs. (**a**–**d**) The fluorescent images of the cytoskeleton staining of HAECs in chamber 1 (**a**), chamber 2 (**b**), chamber 3 (**c**) and sham (**d**), respectively. (**e**–**h**) The length of newly induced filopodia in each chamber at different field intensities calculated from F-actin fluorescence images. Scale bar in (**a**–**d**): 10 µm. Scale bar in insert: 5 µm.

To explore the morphological responses and the RF-EMF exposure, the results of cytoskeleton stained with Phalloidin-TRITC are presented in Figure 3. After continuous exposure for 0.5 h, the new and finger-like cell filopodia in the spreading leading edges of the HAECs was detected (Figure 3a–c). Obviously, the number and length of de novo synthesis filopodia varied under different field intensities. Basically, with the increased intensity, the number and length of new filopodia increased as well, verifying that the field intensity strongly affected the formation of new filopodia. By contrast, in Figure 3d, the leading edges of the cells was almost smooth and flat without RF-EMF exposure, confirming that the field directly contributed to the changes in cell morphology. Indeed, after the RF-EMF exposure, the newly formed filopodia led to morphology variation and actin-driven cell motility, which play an important role in cell migration, initiating cell contacts and cell–cell signal transmission [30]. Furthermore, to clearly analyze the relation between newly formed filopodia and the field intensity, the results of quantitative analyses at different SAR values are shown in Figure 3e–h. According to the fitting curve, the peak appeared when the SAR value was 5.69 W/kg, which suggested the length of filopodia varied between 3.2 µm and 4.0 µm (Figure 3e). Similarly, the length of filopodia at 1.61 W/kg ranged from 2.7 µm to 3.1 µm (Figure 3f). However, the length of filopodia evidently significantly reduced at lower SAR values of 0.66 W/kg and 0 W/kg, which was shorter than 2.0 µm (Figure 3g–h). Consequently, the new filopodia under different field intensities led to the morphological differences.

### 3.4. The Concentration Variation of Cytosolic Ca^2+^ under Gradient RF-EMF

**Figure 4:**
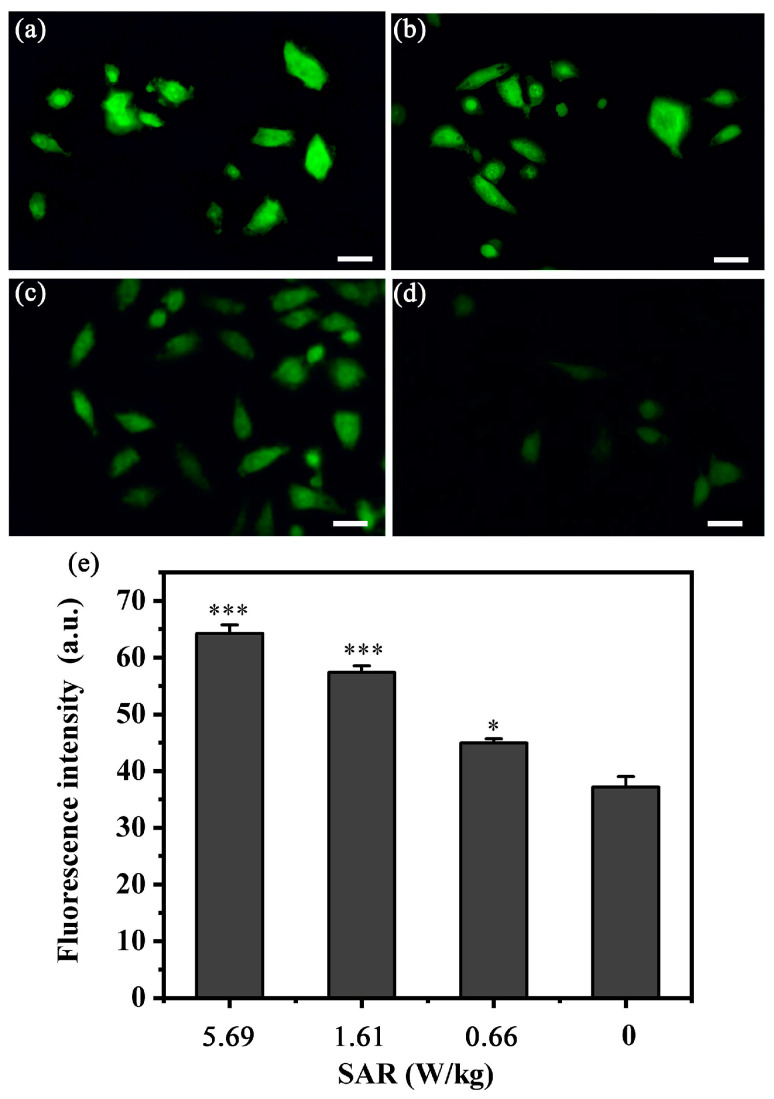
The fluorescent images and fluorescence intensity of cytosolic Ca^2+^ concentration. Cytosolic Ca^2+^ concentration in chamber 1 (**a**), chamber 2 (**b**), chamber 3 (**c**) and sham (**d**), respectively, and the average fluorescence intensity (FI) (**e**). *p* < 0.05 (*); *p* < 0.001 (***) as compared with Sham. Scale bar in (**a**–**d**): 20 µm.

As Ca^2+^ influx caused a rapid increase in cytosolic Ca^2+^ concentration, which resulted in calcium-related bio-responses, particularly in cytoskeletal actin reorganization and damage [31]. To investigate the morphological response mentioned above, the cytosolic Ca^2+^ concentration was analyzed via immunofluorescence assays using a Fluo-4/AM fluorescence probe (Figure 4a–d). To further assess the difference of cytosolic Ca^2+^ concentration in different field intensities, the average fluorescence intensity (FI) was calculated using the software ImageJ (Figure 4e), in which the average FI values of different SAR values were located at 64.25, 57.37, 44.93 and 37.69, respectively. These results indicated that the stronger RF-EMF promoted Ca^2+^ circulation, which induced a higher concentration of cytosolic Ca^2+^, confirming the hypothesis that cytosolic Ca^2+^ concentration was proportional to the field intensity.

### 3.5. Inhibitory Effects of the Cytoskeleton Reorganization

**Figure 5:**
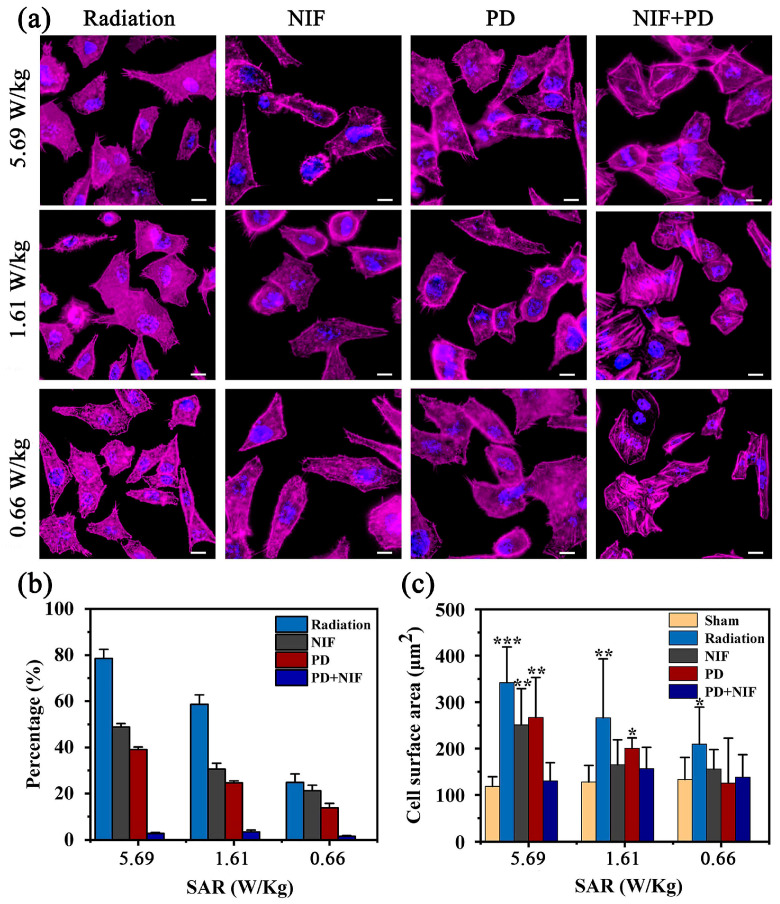
The fluorescent images and quantitative analysis results. (**a**) The fluorescent images of HAECs cytoskeletons pretreated with inhibitors at different field intensities. (**b**) The percentage of actin polymerization in different field intensities, respectively. (**c**) Cell surface area of HAECs pretreated with inhibitors at different field intensities. *p* < 0.05 (*); *p* < 0.01 (**); *p* < 0.001 (***). Scale bar in (**a**): 10 µm.

To further reveal how a cell regulates its cytoskeleton reorganization after exposure, inhibitory experiments of the cytoskeleton reorganization were developed. The HAECs pre-treated with two special inhibitors, ion channel inhibitors and tyrosine kinase inhibitors, were tested to reveal the field-related downstream signaling pathway when cells were exposed to RF-EMF. Figure 5 shows the results and relevant analysis of HAECs treated with inhibitors prior to field exposure. From cytoskeleton staining, it was worth noting that the number and length of the newly formed filopodia gradually decreased, indicating that Nifedipine blocked the L-type voltage-gated calcium channel. In addition, despite the inhibition of Nifedipine, a few newly formed filopodia can be observed, revealing the incomplete inhibition of Nifedipine on the field-related cytoskeleton reorganization. These results also suggested the inhibition of Nifedipine was related to the field intensity. Meanwhile, it was also observed in Figure 5a that the inhibitory effects of cytoskeleton reorganization after pre-treating with PD was somehow similar to those of the inhibition of Nifedipine. In general, with the decrease in the field intensity, the number of newly formed filopodia in all exposure conditions presented a downward trend, but it was noteworthy that there were still some newly formed filopodia in the exposure chambers. Therefore, the PD could not totally inhibit the cytoskeleton reorganization induced by RF-EMF. Additionally, Figure 5a also presents the cytoskeleton reorganization resulting from the synergistic effect of two inhibitors. Evidently, in the presence of gradient field intensity, there were no obvious morphological responses in all exposed chambers. Simultaneously, the smooth edges and flat surfaces of the exposed cells were observed, confirming that the cytoskeleton reorganization was completely inhibited through pre-treatment with these two inhibitors. In conclusion, the aforementioned results of single inhibitors or their combination effects revealed that a single inhibitor, either PD or Nifedipine, partly inhibited actin assembly and the synergistic effect of two inhibitors completely inhibited the cytoskeleton reorganization, which uncovered that both EGFR tyrosine kinase and the L-type voltage-gated calcium channel play important roles in RF-EMF-induced cytoskeleton reorganization.

It is well-known that the newly formed filopodia as a key factor is associated with cell migration and metastasis [32]. Generally, field-induced responses involving actin cytoskeleton reassembly will inevitably result in morphological reorganization, which can be directly investigated by the percentage of actin polymerization (ratio of cells with actin reassembly to total number of cells) and the surface area of cells. In Figure 5b, the ratio of the actin polymerization decreased with the decrease in RF-EMF intensity. Furthermore, it was found that there was a similar reduction between the Nifedipine- and PD-treated groups in different RF-EMF intensities. Especially when involved in their combination effect, a sharp reduction was observed. These results verified the underlying mechanism of field intensity and inhibitory effects affecting actin polymerization. In Figure 5c, average cell surface area was calculated using the software ImageJ. It was found that a significant difference in the cell spreading area between RF-EMF exposure and sham (no-field) can be observed. Detailed data regarding the cell surface area indicated that, compared with sham groups, an obvious increase in surface area can be inspected under the different SAR values, by 64.13% for 5.69 W/kg, 51.06% for 1.61 W/kg and 36.21% for 0.66 W/kg, respectively. Moreover, at the highest SAR value of 5.69 W/kg, when the cells were treated with the special inhibitors prior to RF-EMF exposure, a reduction in the surface area of the cell can be observed in Figure 5c for 26.56% with Nifedipine, for 23.89% with PD and for 62.03% with their combination. A similar downward variation was also observed with the SAR value of 1.61 W/kg, in which the surface area of the cell decreased by 34.59%, 27.69% and 41.24%, respectively. By contrast, at the lowest SAR value of 0.66 W/kg, there was no obvious difference between pre-treatment with the special inhibitors and the sham group. In conclusion, when treated with inhibitors prior to field exposure, either a single inhibitor or their combination, a sharp reduction was observed, revealing the role of special inhibitors in cytoskeleton-related responses to environmental stimuli.

### 3.6. Inhibitory Effects of Ca^2+^ Circulation

**Figure 6:**
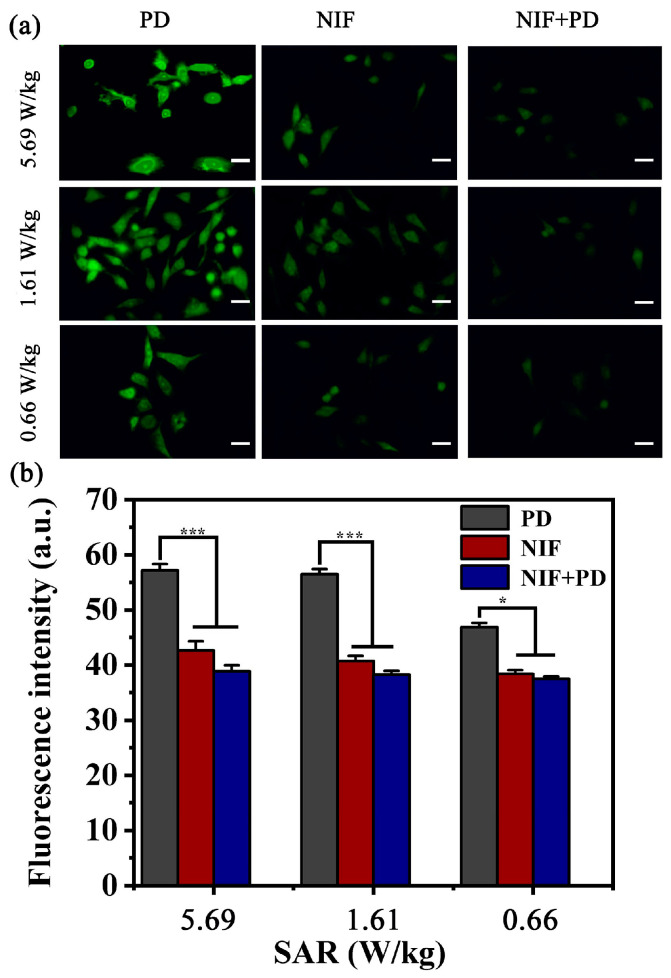
(**a**) The fluorescent images of cytosolic Ca^2+^ staining pretreated with special inhibitors. (**b**) The corresponding fluorescence intensity (FI) of fluorescent images in different chambers. *p* < 0.05 (*); *p* < 0.001 (***). Scale bar in (**a**): 20 µm.

Figure 6 reveals the field-induced variation of cytosolic Ca^2+^ concentration and the relevant downstream signaling pathway when cells were pre-treated with inhibitors in all RF-EMF exposed chambers. In Figure 6a, the fluorescent images of cytosolic Ca^2+^ concentration are presented after being treated with PD, Nifedipine or their combination prior to RF-EMF exposure. No obvious inhibition can be observed when pre-treated with PD. In comparison, the Ca^2+^ mobilization was evidently blocked by the inhibitory effects of Nifedipine. Meanwhile, similar inhibitory effects can be also observed in the group pretreated with the combination of PD and Nifedipine, which is consistent with the fact that RF-EMF can evoke Ca^2+^ influx through the ion channels [16]. Furthermore, the fluorescence results of Ca^2+^ mobilization are shown in Figure 6b. The average FI values for cells pre-treated with PD can reach 57.15, 56.48 and 46.88 at the SAR values of 5.69 W/kg, 1.61 W/kg and 0.66 W/kg, respectively. Compared with PD, the intensity of cells pretreated Nifedipine was as low as 43.63, 42.19 and 38.92, respectively. Similarly, when pre-treated with their combination, there was no significant difference in all exposure groups. These results further reveal the involvement of Ca^2+^ influx in response to the RF-EMF stimulation through the L-type voltage-gated calcium channel.

### 3.7. RF-EMF Induces Cell Alignment

**Figure 7:**
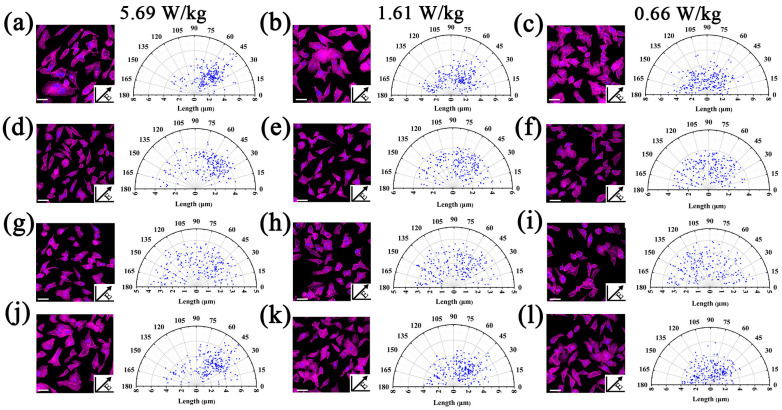
The RF-EMF-induced cell motility. The fluorescent images and polar plots of cytoskeleton alignment of cells after RF-EMF exposure in different chambers, respectively. The insets show the direction of the RF-EMF field. (**a**–**c**): The cytoskeleton alignment after 0.5 h RF-EMF exposure. (**d**–**f**): Time-dependent response of cytoskeleton alignment after recovery for 0.5 h. (**g**–**i**): Time-dependent response of cytoskeleton alignment after recovery for 1.5 h. (**j**–**l**): The cytoskeleton alignment after 0.5 h RF-EMF re-exposure. Polar plots of cytoskeleton alignment (angular coordinate) and elongation (radial coordinate) of the cytoskeleton alignment (each data point represents a cytoskeleton of a cell). Scale bar in (**a**–**c**): 10 µm.

Previous studies have reported that external field stimulation inherently regulated the cells’ alignment, elongation and even the migration [33]. In this work, the differences of cell mobility under different exposure conditions were investigated, including exposure, post exposure and re-exposure. (In each experiment, cells were prepared and treated with the same protocols for exposure as indicated. The resultant samples were incubated with 5% CO_2_ at 37 °C. The resultant effects on cell alignment were collected at 0 h, 0.5 h and 1.5 h after 0.5 h exposure, as well as 0 h after 0.5 h re-exposure, respectively). Figure 7a–c show the results of cell mobility and the quantitative analysis after the first RF-EMF exposure (0.5 h), which uncovers the alignment along the guided channels affected by the certain field direction. Furthermore, as the field intensity increased, more cells intended to align along the guided channels. By contrast, in Figure 7d–f, it was observed that the above cell alignment gradually disappeared when the RF-EMF was repealed. After 1.5 h recovery, cells become randomly distributed even in the samples pre-exposed under the highest field strength (Figure 7g–i). Finally, Figure 7j–l reveal the cell alignment after the second RF-EMF exposure for 0.5 h. After this re-exposure, cells aligned along the guided channel again, which is consistent with the first exposure (Figure 7a–c). These results suggest that the influence of the RF-EMF on cells is likely eliminable and recoverable, but not permanent.

**Figure 8:**
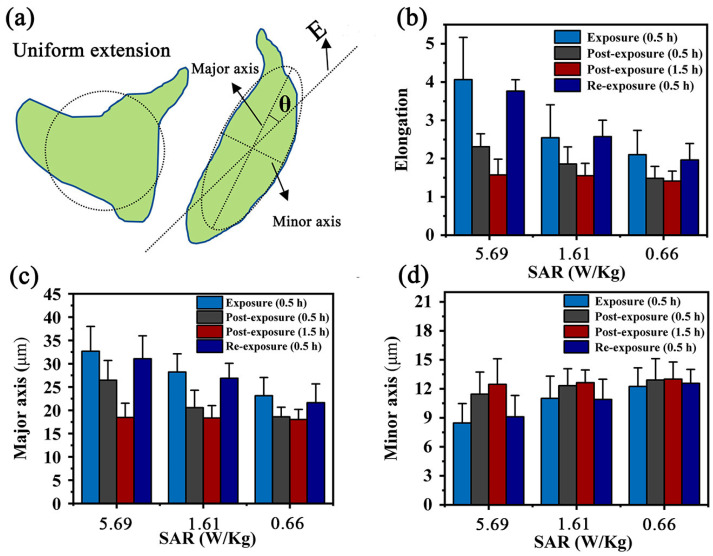
The quantitative analysis results of cell motility. (**a**): Quantification methods for the cell alignment and elongation effects. (**b**–**d**): Elongation values, length and width after EMF exposure. Cells were considered aligned if the angle between the cell’s major axis and the field direction was less than 10°.

To further evaluate cell alignment and morphological reorganization induced by field exposure, the quantitative analysis is shown in Figure 8. The cell area fitted with an ellipse and the angle (*θ*) of the major axis relative to the field direction was performed to facilitate the assessment of alignment (Figure 8a). Similarly, to achieve elongation evaluation, the elongation value was defined as the ratio of the ellipse between the major axis and minor axis. Cells were considered aligned if the angle between the cell’s major axis and the field direction was less than 10°. Figure 8b–d show cell elongation under field exposure, length of the major axis and length of the minor axis of cells, respectively. These results suggested that the field inherently affected cell spreading and elongation. With the increase in field intensity, cells elongated along the guided channel direction instead of spreading all around (Figure 8b). It was obvious that the direction of cell elongation along the guided channel disappeared gradually due to the increase in recovery time. However, cell alignment appeared after the 0.5 h re-exposure. Furthermore, as shown in Figure 8c, after 1.5 h recovery, the cell length decreased to 16 μm, but significantly increased to 32 μm after re-exposure for 0.5 h. By contrast, cell width demonstrated a completely opposite trend, varying from 12 to 8 μm (Figure 8d). These above results further verified the recoverability of RF-EMF exposure and eliminated the public concern about the potential health hazard of RF-EMF exposure. It should be noted that each experiment was repeated at least three times, aiming to ensure the consistency of results.

## 4. Conclusions

In this work, a versatile and novel electromagnetic radiation assessment system integrated with a microfluidic chip was designed, aiming to provide a highly effective, easily-operated and reliable strategy to assess the possible health risks of RF-EMF exposure in vitro. We developed the cell radiation experiments in vitro to validate the practicability of our setup. With continuous exposure for 0.5 h, the obvious growth of new protrusions in the spreading leading edge and morphology variation at different field intensities were detected. Meanwhile, the difference in surface area and the actin polymerization between multiple field intensities was investigated, confirming the RF-EMF-induced morphological response. Additionally, the intracellular Ca^2+^ concentration was detected using immunofluorescence staining, showing the trend that the intracellular Ca^2+^ concentration increased with field strength. To unveil the mechanism of morphological response induced by RF-EMF exposure, the inhibitory assays were performed by pre-treatment with specific inhibitors such as calcium channel inhibitor (Nifedipine) and tyrosine kinase inhibitor (PD). It was revealed that the cell morphological response, including newly formed protrusion patterns, changes in cellular shapes and the Ca^2+^ influx, were inhibited through the effects of inhibitors, confirming the role of the phosphorylation of tyrosine kinase and the L-type voltage-gated calcium channel in cell morphological response. Additionally, after exposure, with the increase in field intensity, cells could be more easily elongated and aligned along the designed guided channel direction instead of spreading all around. Finally, to further eliminate the public concern about potential health hazards of RF-EMF exposure, these assays aiming for exploring the recoverability after RF-EMF exposure and secondary response after re-exposure were developed. The relevant results suggested that the state of the cell could recover the state before RF-EMF exposure and the influence of re-exposure on cells is consistent with that of the first exposure. Compared with traditional RF radiation devices, the further development of this exposure system will be able to meet the need of the operability of setup, the precision of in situ detection and the high-throughput analysis of cell response to RF-EMF.

## Data Availability

The data presented in this study are available on reasonable request from the corresponding author.

## Supplementary Materials

The following supporting information can be downloaded at: https://www.mdpi.com/article/10.3390/bios13080763/s1, Figure S1: Fabrication process of the microfluidic device by soft-lithography; Figure S2:The experimental setup of the microfluidic device. Figure S3: The selection position of simulated EMF in a single chamber. Figure S4: The user interface of exposure control system.
