# Supplementary material for: A Novel and Versatile Microfluidic Device for Cell Assays under Radio Frequency Exposure
Source: Biosensors (Basel). 2023 Jul 27;13(8):763. doi: 10.3390/bios13080763 (PMC10452282; doi:10.3390/bios13080763)
Supplement: Supplementary file 1 [file biosensors-13-00763-s001.zip › biosensors-2362284-supplementary.pdf]

biosensors

MDPI

Supporting information

A novel and versatile microfluidic device for cell mobility analysis under radio frequency exposure

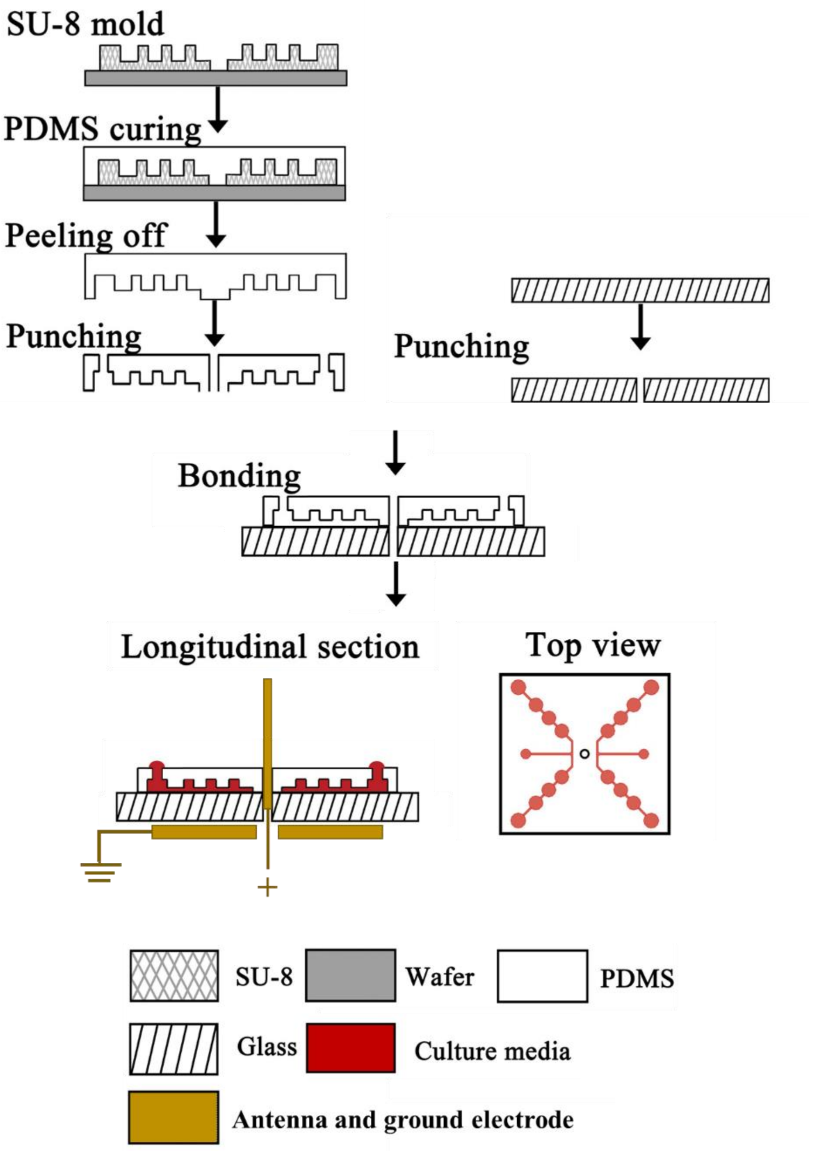

Figure S1. Fabrication process of the microfluidic device by soft-lithography. After photolithography, the PDMS casting is manually aligned and bonded on the glass substrate with a hole punched for antenna. A copper wire is embedded inside 10 : 1 PDMS before curing to ensure the antenna position. After plasma bonding with the punched PDMS casting, ground electrode was placed on bottom of the microdevice. Both the antenna and the ground electrode were connected with RF source with cables.

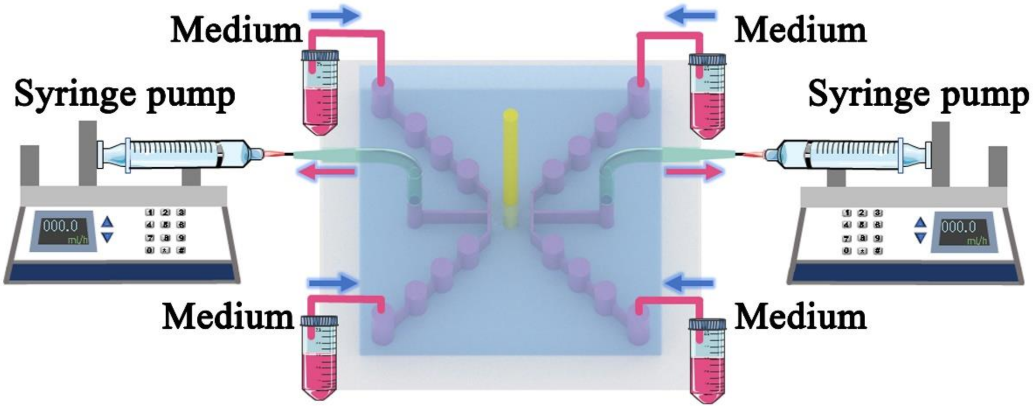

Figure S2. The experimental setup of the microfluidic device.

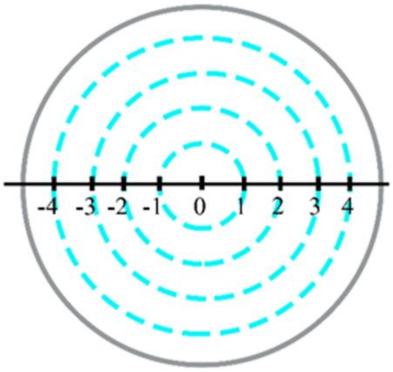

Figure S3. The selection position of simulated EMF in a single chamber.

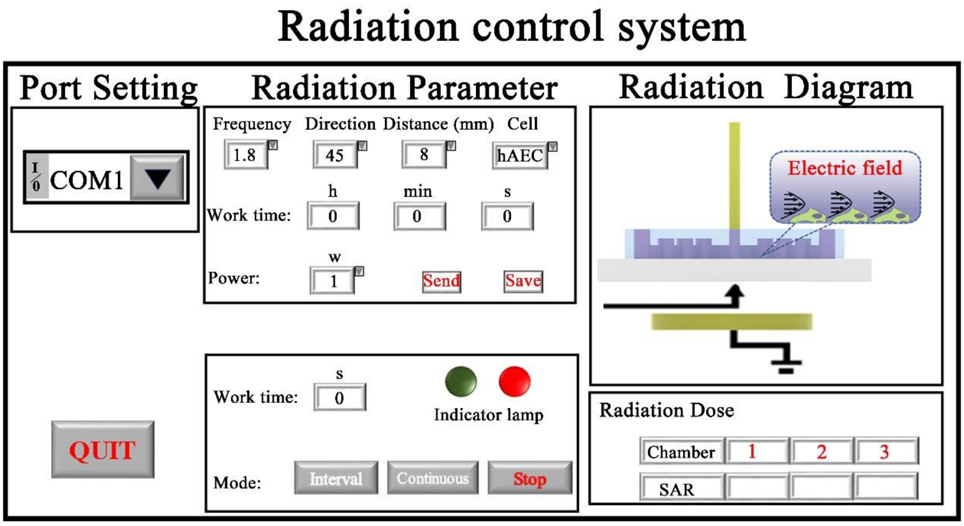

Figure S4. The user interface of exposure control system.
